# Institutional Notes and News

**Published:** 1911-07-01

**Authors:** 


### Institutional Notes and News.

**Noteworthy Extension at St. Leonards.**

We are authoritatively informed that owing to the long waiting-list, the committee of the Eversfield Hospital for Consumption, St. Leonards-on-Sea, decided last year to increase the accommodation by adding from twenty-five to thirty beds and making certain necessary alterations and additions at an estimated cost of £7,500. This sum represented £2,000 for extra lavatory accommodation, baths, and so forth; £2,000 for widening the balconies, new wards, sitting-rooms for men and women, and for enlarging the dining-hall, all of which are to have a south aspect facing the sea; and £3,500 for nurses' home, dispensary, and laboratory on the recently purchased plot of land opposite the hospital. As the various appeals resulted in upwards of £1,500 being obtained, the committee decided at once to commence the first portion of the work, and the sanitary block was started a few days ago. It is hoped that the whole scheme will be completed before the close of next year. Mr. John Payne, A.R.I.B.A., of London, is the architect in charge of the alterations.

**New Home for Women at Charnwood.**

Mr. S. A. Gimson (Vice-President) occupied the chair at the laying of the commemoration stones of the new Convalescent Home for Women at Woodhouse Eaves on Saturday last. General regret was expressed at the absence through ill-health of Mr. Orson Wright, J.P. (one of the trustees of the late Mr. Edward Higgs, under whose will the building of the Home was made possible). The architects (Messrs. Scale and Riley, A.R.I.B.A.) having presented Mr. Wm. Newbury, the remaining executor, with an inscribed silver trowel, that gentleman declared the first commemoration stone to be well and truly laid. Mr. Gimson then asked Sir Edward Wood, J.P., the President of the Society, to lay the second stone. As we noted in last week's Hospital, a more romantic site could not have been chosen. On one of the highest points of Charnwood Forest, the grounds are nine acres in extent, and the Home overlooks an expanse of rugged and woody country. The grounds have been retained in their natural state, and the huge granite boulders in the declivities are reminiscent of the Ice Age. The Home itself will contain forty-two bedrooms, dining and reading-rooms, with accommodation for the matron and staff, and laundry. Gas is provided and water from the Leicester Corporation mains. The Home is expected to be ready next Easter. The builders are Messrs. Cole, of Leicester. Through the kindness of Mr. J. Gibson Clarke (Vice-President and Chairman of the Finance Committee of the Society) and Mrs. Clarke the guests were invited to tea in the grounds. The Society, with the opening of the present Home, will have provided Convalescent Homes for both male and female members, and should funds permit a consumptive sanatorium will be provided. Membership of the Society is granted to subscribing men and women for ½d. a week for those earning 10s. a week and under and 1d. per week for those earning upwards of 10s. The total amount received from these contributions last year was over £13,000, 70 per cent. of which is contributed to the Leicester Infirmary, the remainder going to the convalescent work of the Society. Mr. H. H. Woolley is the Secretary, and the amount of his work may be guessed from the extent of the Society's activities.

**Women's Work at Cardiff Infirmary.**

A most successful meeting was held the other day in connection with the Cardiff Infirmary. This was the annual meeting of the Ladies' Needlework Guild and Linen Association, which resolved itself, after the business proceedings were finished, into a garden party in the Infirmary grounds. Mrs. Hughes, the wife of the Bishop of Llandaff, presided over the meeting, and emphasised the value to the organisation of individual effort. This was aptly illustrated by the reports of the presidents of the Guild, Mrs. Lewis announcing that 473 garments for patients had been received by the Needlework Guild, while Mrs. Lynn Thomas reported that 1,382 articles of linen had been received during the year. No linen had had to be bought since the Association had been in existence. The rule of the Guild is that each associate shall supply at least two articles each year. Colonel Bruce Vaughan took advantage of the occasion to make a powerful appeal for the Cardiff City Memorial to King Edward VII., so that the new wing of the Infirmary might be opened without delay. This much-needed accommodation is now practically completed, but an additional £4,000 per annum is yet required for its maintenance, while there are nearly 800 patients—men, women, and children—waiting for admission. "There are," said Colonel Vaughan, "254 children waiting at present. Last month we admitted 27, 19 of whom were emergencies—burns and street accidents—thus reducing the waiting-list only by eight, and probably as many as that have since applied for admission. We have only 34 children's beds in the old building." The subsequent tea on the children's lawn was enlivened by a programme of music from the Cardiff Post Office Band. There were over five hundred ladies present. The Board of Management, at a meeting on the following day, heartily approved of a happy suggestion emanating from Colonel Bruce Vaughan that one of the large wards in the new wing, the walls of which are ready for the finishing touches, should be tiled with pictures illustrative of the Coronation and of the investiture next month at Carnarvon of H.R.H. the Prince of Wales.

**A New Sanatorium School.**

Mr. W. F. D. Smith, Chairman of King's College Hospital, has issued an appeal for the children's department of the Maitland Sanatorium, Peppard Common, Oxford, in the course of which he says: "Kindercot, as the Sanatorium School is called, meets the long-felt need of combining a thorough education with the open-air treatment essential for children who are suffering from incipient consumption, who are debarred from attending all the ordinary schools, and whose education, at the most important period of their lives, is consequently at a standstill. The experiment has proved even more successful than was anticipated. The children all respond to treatment in such a remarkable manner, and improvement takes place so rapidly, that almost at once they are able to avail themselves of the education provided. A special feature is made of outdoor lessons, and the farm provides an excellent opportunity of becoming acquainted with country life. The results have been so excellent that the committee is making a special effort to raise the necessary funds to extend Kindercot, and they think it would be a good way to commemorate the year of the Coronation to complete the building, which when finished will accommodate twenty children. For this purpose a sum of £1,500 is required."

